# Effect of ploidy, recruitment, environmental factors, and tamoxifen treatment on the expression of sigma-2 receptors in proliferating and quiescent tumour cells

**DOI:** 10.1038/sj.bjc.6690789

**Published:** 1999-11

**Authors:** I Al-Nabulsi, R H Mach, L-M Wang, C A Wallen, P C Keng, K Sten, S R Childers, K T Wheeler

**Affiliations:** Department of Radiation Oncology, Radiology andPhysiology and Pharmacology, Wake Forest University School of Medicine, Medical Center Boulevard, Winston-Salem, NC 27157, USA; Cancer Center, University of Rochester Medical Center, Rochester, NY 14642, USA

**Keywords:** sigma-2 receptors, proliferative tumour cells, quiescent tumour cells, biological factors, physiological factors

## Abstract

Recently, we demonstrated that sigma-2 receptors may have the potential to be a biomarker of tumour cell proliferation (Mach et al (1997) *Cancer Res***57**: 156–161). If sigma-2 receptors were a biomarker of tumour cell proliferation, they would be amenable to detection by non-invasive imaging procedures, thus eliminating many of the problems associated with the flow cytometric measures of tumour cell proliferation presently used in the clinic. To be a good biomarker of tumour cell proliferation, the expression of sigma-2 receptors must be essentially independent of many of the biological, physiological, and/or environmental properties that are found in solid tumours. In the investigation reported here, the mouse mammary adenocarcinoma lines, 66 (diploid) and 67 (aneuploid), 9L rat brain tumour cells, and MCF-7 human breast tumour cells were used to study the extent and kinetics of expression of sigma-2 receptors in proliferative (P) and quiescent (Q) tumour cells as a function of species, cell type, ploidy, pH, nutrient depletion, metabolic state, recruitment from the Q-cell compartment to the P-cell compartment, and treatment with tamoxifen. In these experiments, the expression of sigma-2 receptors solely reflected the proliferative status of the tumour cells. None of the biological, physiological, or environmental properties that were investigated had a measurable effect on the expression of sigma-2 receptors in these model systems. Consequently, these data suggest that the proliferative status of tumours and normal tissues can be non-invasively assessed using radiolabelled ligands that selectively bind sigma-2 receptors. © 1999 Cancer Research Campaign


British Journal of Cancer (1999) 81(6), 925–933
© 1999 Cancer Research Campaign
Article no. bjoc.1999.0789

### Effect of ploidy, recruitment, environmental factors, and tamoxifen treatment on the expression of sigma-2 receptors in proliferating and quiescent tumour cells

**I Al-Nabulsi[1], RH Mach[2,3], L-M Wang[2], CA Wallen[2], PC Keng[4], K Sten[2], SR Childers[3] and KT Wheeler[1,2]**

Department of [1]Radiation Oncology, [2]Radiology and [3]Physiology and Pharmacology, Wake Forest University School of Medicine, Medical Center Boulevard, Winston-Salem, NC 27157, USA; [4]Cancer Center, University of Rochester Medical Center, Rochester, NY 14642, USA

**Summary** Recently, we demonstrated that sigma-2 receptors may have the potential to be a biomarker of tumour cell proliferation (Mach et al (1997) *Cancer Res* **57**: 156–161). If sigma-2 receptors were a biomarker of tumour cell proliferation, they would be amenable to detection by non-invasive imaging procedures, thus eliminating many of the problems associated with the flow cytometric measures of tumour cell proliferation presently used in the clinic. To be a good biomarker of tumour cell proliferation, the expression of sigma-2 receptors must be essentially independent of many of the biological, physiological, and/or environmental properties that are found in solid tumours. In the investigation reported here, the mouse mammary adenocarcinoma lines, 66 (diploid) and 67 (aneuploid), 9L rat brain tumour cells, and MCF-7 human breast tumour cells were used to study the extent and kinetics of expression of sigma-2 receptors in proliferative (P) and quiescent (Q) tumour cells as a function of species, cell type, ploidy, pH, nutrient depletion, metabolic state, recruitment from the Q-cell compartment to the P-cell compartment, and treatment with tamoxifen. In these experiments, the expression of sigma-2 receptors solely reflected the proliferative status of the tumour cells. None of the biological, physiological, or environmental properties that were investigated had a measurable effect on the expression of sigma-2 receptors in these model systems. Consequently, these data suggest that the proliferative status of tumours and normal tissues can be non-invasively assessed using radiolabelled ligands that selectively bind sigma-2 receptors. © 1999 Cancer Research Campaign

**Keywords:** sigma-2 receptors; proliferative tumour cells; quiescent tumour cells; biological factors; physiological factors

For years, the proliferative status of a tumour has been known to have a profound effect on the outcome of both chemotherapy and radiotherapy treatments (Shackney et al, 1978; McGuire and Dressler, 1987; Chaval et al, 1989; Grogan et al, 1989; Hedley et al, 1993*a*, 1993*b*; Begg, 1995). For example, both laboratory and clinical data suggest that accelerated and/or hyperfractionated radiotherapy schedules are better for the treatment of rapidly proliferating tumours, while conventional daily radiotherapy schedules are better for the treatment of slowly proliferating tumours (e.g. Thames et al, 1983; Awwad, 1992; Begg et al, 1992; Begg, 1995; Corro et al, 1995; Sarkaria et al, 1995). Similarly, rapidly proliferating tumours respond better to cell cycle-specific chemotherapeutic agents (e.g. 5-fluorouracil, 1-β-D-arabinofuranosylcytosine (Ara-C), etc.), while slowly proliferating tumours respond better to cell cycle-non-specific chemotherapeutic agents (e.g. 1,3 bis-(2-chloroethyl)-1-nitrosourea (BCNU), cisplatin (Cis-DDP), etc). Consequently, biomarkers of tumour cell proliferation, particularly those that could be detected with non-invasive imaging techniques, should be very useful in selecting agents and/or schedules for treating a patient's tumour.

*Received 3 November 1998*
*Revised 28 April 1999*
*Accepted 10 May 1999*

*Correspondence to:* KT Wheeler, Department of Radiology, Wake Forest University School of Medicine, Medical Center Boulevard, Winston-Salem, NC 27157, USA

To date, flow cytometric measurements of various cell cycle parameters or biomarkers (e.g. S-phase fraction, proliferating cell nuclear antigen (PCNA), Ki-67, potential doubling time ($T_{pot}$), etc.) have been the most extensively studied predictors of tumour cell proliferation (Hedley et al, 1993*a*, 1993*b*; Begg, 1995). However, these flow cytometric predictors of outcome have met with limited success in selecting anti-tumour agents or treatment schedules for individual patients due to sampling and technical problems (Dressler, 1993; Hedley et al, 1993*b*; Begg, 1995; Haustermans et al, 1995; Tsang et al, 1995). Recently, we have been studying sigma-2 receptors as possible biomarkers of tumour cell proliferation because these receptors are potentially amenable to detection by non-invasive imaging techniques (e.g. positron emission tomography and single-photon emission tomography). Using the well-characterized in vitro mouse mammary adenocarcinoma model, line 66, proliferative (P) cells were shown to express ≈10 times more sigma-2 receptors per cell than quiescent (Q) cells (Mach et al, 1997). In addition, the kinetics for the loss of sigma-2 receptors from 66 Q-cells was identical to the kinetics for the loss of PCNA from 66 Q-cells. However, these results were obtained with a single rodent tumour cell line, so any suggestion that sigma-2 receptors might be used as a universal biomarker of tumour cell proliferation requires further investigation.

In the study presented here, several in vitro tumour models were employed to test the hypothesis that the expression of sigma-2 receptors is predominantly influenced by a tumour cell's ability to progress through the cell cycle in a timely manner, and not by other biological, physiological, or environmental properties of the tumour. Using mouse mammary adenocarcinoma cells, lines 66 and 67, 9L rat brain tumour cells, and MCF-7 human breast tumour cells, the extent and kinetics of expression of sigma-2 receptors in P and Q tumour cells was studied as a function of species, cell type, ploidy, pH, nutrient depletion, metabolic state, recruitment from the Q-cell compartment to the P-cell compartment, and treatment with tamoxifen. The data suggest that the expression of sigma-2 receptors may indeed be a universal biomarker of tumour cell proliferation, assuming that these in vitro results can be duplicated in solid tumour models.

### MATERIALS AND METHODS

### Drugs

DTG (1,3-di-*o*-tolyguanidine) and (+)-pentazocine were purchased from Research Biochemical International (Natick, MA, USA). [$^3$H]DTG was purchased from Dupont-NEN (Billerica, MA, USA). Tamoxifen was purchased from Sigma Chemical Company (St. Louis, MO, USA).

### Cell culture procedures

The 66 (diploid) and 67 (aneuploid) cells in these experiments were originally derived from a mouse mammary adenocarcinoma and cultured in Waymouth's medium supplemented with 3% fetal calf serum, 6% newborn calf serum, 6% horse serum, 1% glutamine, 80.5 mg ml$^{-1}$ of streptomycin and 80.5 units ml$^{-1}$ of penicillin as previously described (Wallen et al, 1984*a*, 1984*b*). The 9L rat brain tumour cells were grown in BME supplemented with 10% newborn calf serum, 1% glutamine, 80.5 mg ml$^{-1}$ of streptomycin and 80.5 units ml$^{-1}$ of penicillin as previously described (Wallen et al, 1980; Wheeler et al, 1992). MCF-7 cells were grown in phenol red-free essential modified Eagle's medium (EMEM) supplemented with 10 µg ml$^{-1}$ of bovine insulin, 1% glutamine, 1% nonessential amino acids, 80.5 mg ml$^{-1}$ of streptomycin, 80.5 units ml$^{-1}$ of penicillin and 5% calf serum that had been treated with dextran-coated charcoal for 45 min at 55°C to remove any endogenous hormones (Eckert and Katzenellenbogen, 1982; Katzenellenbogen et al, 1984; Berthois et al, 1986). All cells were grown at 37°C in a 5% carbon dioxide atmosphere. At 3- to 6-month intervals, each cell line was rejuvenated from frozen stock and tested for *Mycoplasma* (Mach et al, 1997).

With the exception of the recruitment experiments, exponentially growing cells were trypsinized from stock cultures and seeded at: (a) $1–2 \times 10^5$ cells per 25-cm$^2$ flask in 5 ml of the appropriate medium for the growth curve experiments, and (b) $1–2 \times 10^6$ cells per 175-cm$^2$ flask in 30 ml of the appropriate medium for the sigma-2 receptor experiments. For the growth curve experiments, three flasks were trypsinized at various times after seeding, the cells were counted and sized on a Coulter counter/channelizer system, and the mean ± 1 s.d. of the three counts was plotted as a function of the incubation time. The doubling time was calculated from a regression analysis of the day 1–4 data. In the sigma-2 receptor experiments, exponentially growing cells were obtained from 17 × 175-cm$^2$ flasks seeded 3 days prior to harvesting, and plateau phase cells were obtained from 12 × 175-cm$^2$ flasks seeded at 5–12 days prior to harvesting, depending on which cell line was used in the experiment.

### Cell proliferation analysis

The percentage of 67, 66 and 9L cells labelled by a short (1 h) or long (36 h) exposure to bromodeoxyuridine (BrdU) ($10^{-5}$ M) was determined flow cytometrically because the in vitro growth conditions in these sigma-2 receptor experiments were slightly different from those described previously (Wheeler and Wallen, 1980; Wallen et al, 1984*a*, 1984*b*). Similar BrdU labelling experiments were not performed for the MCF-7 cells because the in vitro growth conditions were identical to those where the proliferation kinetics had recently been determined by flow cytometrically measuring the Ki-67 and idoxuridine (IdU) labelling index (Dong et al, 1997).

Both unlabelled and BrdU-labelled cultures were handled identically. After trypsinization, the resulting single-cell suspensions were gently centrifuged at 4°C, resuspended in phosphate-buffered saline (PBS), and fixed in 70% ethanol at a final concentration of $1–2 \times 10^6$ ml$^{-1}$. For the flow cytometry analysis, $1.5 \times 10^6$ cells were first incubated for 20 min at 37°C with 0.2 mg ml$^{-1}$ of pepsin in 2 N HCl–PBS, washed twice in PBS containing 0.5% fetal bovine serum (FBS), and then incubated for 45 min with a mouse anti-BrdU antibody conjugated to fluorescein isothiocyanate (Boehringer-Mannheim, Indianapolis, IN, USA). The cells were then washed in 1 ml of PBS containing 0.5% FBS and 0.5% Tween-20, incubated for 30 min in RNAase (1 mg ml$^{-1}$) and stained with propidium iodide (10 µg ml$^{-1}$). All flow cytometry was performed using a Coulter Epics flow cytometer equipped with an air-cooled argon laser using an excitation wavelength of 488 nm. In each experiment, cells isolated from unlabelled 67, 66 and 9L cultures were handled as described above in order to set the gating parameters that compensate for autofluorescence and non-specific binding of the anti-BrdU monoclonal antibody.

Although exponentially growing 67, 66 and 9L tissue culture cells that were pulse-labelled with BrdU contained as much BrdU per cell as exponentially growing human tissue culture cells, the background fluorescence due to autofluorescence and non-specific binding of the monoclonal antibody to the 67, 66 and 9L tumour cells was much higher than that seen with human cells because the commercially available antibody was produced in mice. Consequently, the signal to noise ratio was much less than that normally obtained with human tumour cells. Two gating methods were employed to estimate the percentage of BrdU–labelled cells in each sample. The flow cytometry data was displayed either as a bivarate distribution of the BrdU content as a function of the cell's position in the cell cycle or as a histogram of the BrdU content. Gating parameters were selected so that ≤ 5% of the unlabelled control cells had fluorescence intensities sufficient to be considered BrdU-labelled cells. Although both methods gave identical results, a box analysis on the bivariate distributions was used to generated the data in Table 1.

### Determination of the sigma-2 receptor density

For each cell population, the tissue culture flasks were placed on ice, the medium was removed, the flasks were rinsed with ice-cold medium without serum, and the cells harvested with a plastic scraper (Mach et al, 1997). In each experiment, the cells in three additional 175-cm$^2$ flasks were trypsinized, counted and their protein content determined by the method of Bradford (1976). For the various calculations, it was assumed that the number of scraped cells per flask and their protein content were identical to

 © 1999 Cancer Research Campaign

**Table 1** Summary of the percentage of BrdU-labelled cells obtained when exponential and plateau phase 67, 66 and 9L tissue culture cells were exposed to $10^{-5}$ M BrdU for 1 h or 36 h

| | | BrdU exposure time | | |
|---|---|---|---|---|
| **Cells** | **Days in culture** | **0 h** | **1 h** | **36 h** |
| 67 | 3 | 3.2 | 39.7 | 44.1 |
| | 10 | 1.1 | 4.3 | 6.1 |
| 66 | 3 | 2.1 | 35.1 | 63.0 |
| | 10 | 0.3 | 1.9 | 9.5 |
| 9L | 3 | 3.1 | 16.7 | 89.2 |
| | 5 | 5.0 | 21.2 | 68.1 |

those obtained by trypsinizing the cells. After harvesting, cell membranes were prepared as previously described (Mach et al, 1997), and aliquots containing 1 mg $ml^{-1}$ of protein were stored at –80°C until assayed for sigma-2 receptors.

The sigma-2 receptor density was determined for each membrane preparation as previously described by Mach et al (1997). Briefly, 30–60 µg of protein was incubated with 4 nM [$^3$H]DTG and varying amounts of cold DTG (0.1–1000 nM) in the presence of (+)-pentazocine (100 nM) to mask the sigma-1 sites. Nonspecific binding was determined in the presence of 5 µM DTG. After rapidly harvesting each sample on filters and counting the radioactivity with a liquid scintillation spectrometer, the binding data were analysed with the Scatchard program, EBDA (Biosoft, Miltown, NJ, USA), using the COLD option to calculate the $B_{max}$ values. The sigma-2 receptor density was then calculated as previously described (Mach et al, 1997).

### Experimental design

In order to determine the effect of ploidy on the expression of sigma-2 receptors in P- and Q-cells, 17 flasks of aneuploid mouse mammary adenocarcinoma cells, line 67, were seeded 3 days prior to harvesting 12 flasks of 67 Q-cells. Thus, a 3-day P-cell population could be analysed simultaneously with either a 7-, 10-, or 12-day Q cell population. The P- and Q-cells were harvested by scraping, cell membranes were prepared, and the sigma-2 receptor density was measured as previously described (Mach et al, 1997). The results from the aneuploid 67 cells were compared to the previously published results from the diploid 66 cells to determine if ploidy influenced the expression of sigma-2 receptors in P and Q mouse mammary adenocarcinoma cells.

In order to determine if the expression of sigma-2 receptors followed the kinetics of recruitment of Q-cells back into the P-cell compartment, 66 or 67 cells were trypsinized from 10-day cultures and reseeded at $10^7$ cells per 175-$cm^2$ tissue culture flask (Wallen et al, 1984*b*). The initial sigma-2 receptor density (day 0) was determined on an aliquot of the 10-day Q cells that were used to set the recruitment experiment. On day 2, 4, 7, 10, 14 and 17 after subculturing, the cells were harvested by scraping, cell membranes were prepared, and the sigma-2 receptor density measured as previously described (Mach et al, 1997). The kinetics of the expression of the sigma-2 receptors was then compared with the population growth kinetics as the cells progressed from Q to P and then back to Q.

In order to determine if factors such as cell–cell contact, nutrient depletion, changes in metabolic state, or low pH (6.6–6.8) might be responsible for the loss of sigma-2 receptors in plateau phase cells, 9L rat brain tumour cells were seeded at 2–3 × $10^6$ cells per 175-$cm^2$ flask. These 9L cells grew exponentially for 3–4 days before reaching a plateau on day 5 that results from an equilibrium between the addition of 9L cells to the culture through cell division and loss of 9L cells from the culture through cell death and lysis (Wheeler and Wallen, 1980). On day 5 after subculture, all of these 9L cells appeared healthy, even though a few of these cells were clonogenically dead or dying. By day 7, 50% of the 9L cells were rounded up and in the final stages of cell death because the glucose in the medium had been depleted (Li, 1982*a*, 1982*b*). The clonogenic efficiency of these 7-day 9L cells was < 1%. The sigma-2 receptor density of 3-day 9L cells was 6.3 times greater than the sigma-2 receptor density of these nonclonogenic 7-day 9L cells. Consequently, if the sigma-2 receptor density remained relatively constant for day 3 and day 5 9L cells, cell–cell contact, nutrient depletion, metabolic state and low pH (6.6–6.8) are not likely to have much influence on the expression of sigma-2 receptors in solid tumours.

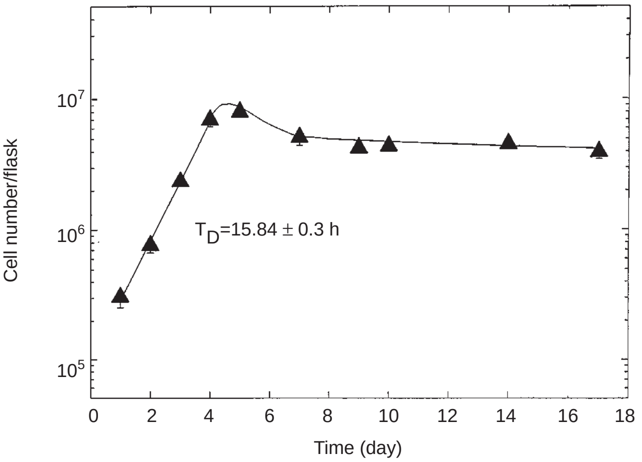

**Figure 1** In vitro growth curve for aneuploid mouse mammary adenocarcinoma cells, line 67. Each data point represents the mean ± 1 s.d. of the cell counts in three independent experiments. If not shown, the error bars lie within the data point. The population doubling time ($T_D$) was determined from a least squares linear regression analysis of the natural log transformation of the day 1–4 data

In order to determine if changes in the sigma-2 receptor density mimic changes in the proliferative status of tumour cells after treatment with chemotherapeutic agents, oestrogen-responsive MCF-7 cells obtained from the American Type Tissue Culture repository were seeded at 2 × $10^6$ cells per 175-$cm^2$ flask and grown for 3 days in phenol red-free medium containing charcoal scrubbed serum (Berthois et al, 1986). A 10 mM stock solution of tamoxifen was prepared in ethanol and stored at –20°C. On day 3, the tamoxifen stock solution was diluted in ethanol and added to the culture medium to produce a final tamoxifen concentration of 1 nM and a final ethanol concentration of 0.1%. In some experiments, the medium containing tamoxifen was removed on day 6, and the cells retreated for another 3 days with fresh medium containing 1 nM tamoxifen. On day 6 and day 9 after seeding, both untreated and tamoxifen treated cells were harvested by scraping, cell membranes were prepared, and the sigma-2 receptor density was determined as previously described (Mach et al, 1997). The reduction in the sigma-2 receptor density measured in these experiments was then quantitatively compared to the reduction in

© 1999 Cancer Research Campaign

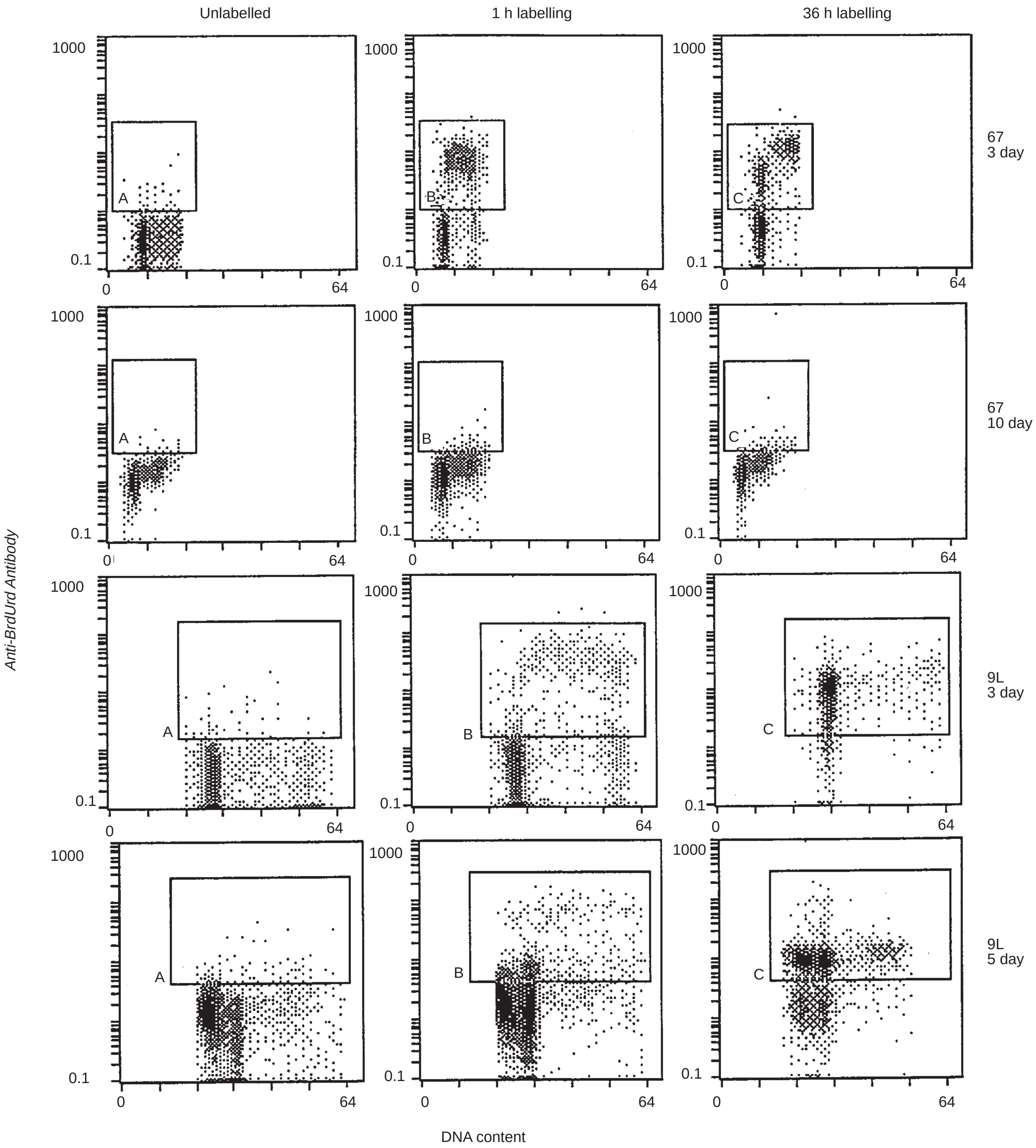

**Figure 2** Flow cytometric determination of the percentage of labelled cells obtained when exponential or plateau phase cultures of 67 and 9L cells were exposed to $10^{-5}$ M BrdU for 1 h or 36 h. Each dot within the box represents a cell that is considered to have been labelled by BrdU and is, therefore, a proliferating cell. The gating parameters were chosen so that ≤5% of a population of cells that had not been exposed to BrdU resided in the box (left panels)

*© 1999 Cancer Research Campaign*

**Table 2** Summary of the $\sigma_2$ receptor analyses for 3-day 67 P-cells and either 7-day, 10-day, or 12-day 67 Q-cells[a]

| | $B_{max}$ (fmol mg$^{-1}$ protein) | Receptors per cell |
|---|---|---|
| 3-day P-cells | 7937 ± 987 | 533 204 ± 178 809 |
| 7-day Q-cells | 4989 ± 1465 | 89 362 ± 29 172 |
| P:Q ratio | 1.66 ± 0.11 | 5.99 ± 0.27 |
| 3-day P-cells | 8652 ± 988 | 348 655 ± 178 809 |
| 10-day Q-cells | 5045 ± 1374 | 51 865 ± 11 706 |
| P:Q ratio | 2.02 ± 0.21 | 7.74 ± 0.64[b] |
| 3-day P-cells | 12 824 ± 1328 | 856 505 ± 243 956 |
| 12-day Q-cells | 6032 ± 2061 | 116 128 ± 67 099 |
| P:Q ratio | 2.36 ± 0.28 | 8.65 ± 1.16[c] |

[a]All values are the mean ± 1 SE from at least three independent experiments. The P-cells experiments were run simultaneously with the corresponding Q-cell experiments. [b]Value statistically different from 7-day P:Q ratio; $P < 0.02$. [c]Value statistically different from 7-day P:Q ratio; $P < 0.03$, but not statistically different from 10-day P:Q ratio; $P > 0.4$.

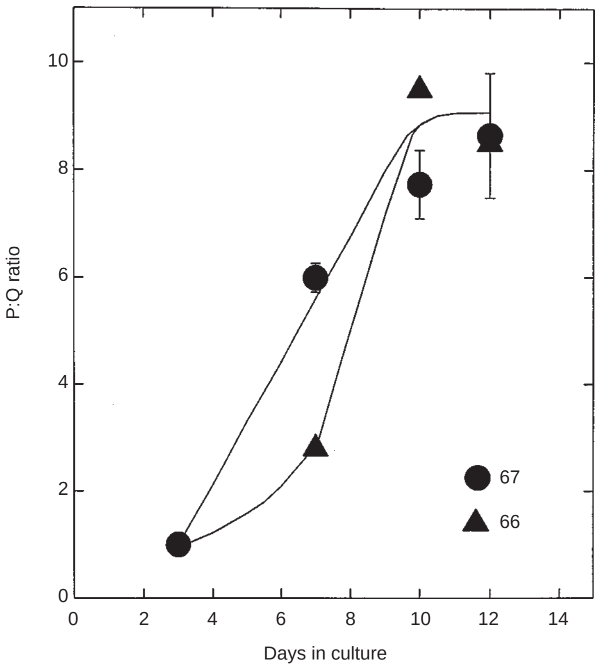

**Figure 3** Ratio of the sigma-2 receptor density in 3-day 66 or 67 P-cells to the sigma-2 receptor density in 7-, 10- or 12-day 66 or 67 Q-cells. Each data point is the mean ± 1 s.d. of the P:Q ratios obtained in three independent experiments. If not shown, the error bars lie within the data point the Ki-67 labelling index, AgNOR scores, and IdU-labelling index recently reported for these MCF-7 cells treated with 4-hydroxy-tamoxifen under identical conditions (Dong et al, 1997).

### RESULTS

### Effect of ploidy on the expression of sigma-2 receptors during the P to Q transition

The 67 cells in these experiments grew exponentially for 4 days with a population doubling time of 15.8 ± 0.3 h (Figure 1). After day 5, there was a slight reduction in the number of cells per flask until a stable plateau phase was reached by day 7. Both the shape of the growth curve and the population doubling time for these 67 cells were identical to those previously published by Wallen et al (1984*a*).

The percentage of 67 cells in day 3 cultures that were labelled by a 1 h pulse of BrdU in 175-cm$^2$ flasks (≈39%) was slightly lower than that expected for an exponentially growing population with a doubling time of 16 h (Figures 1 and 2, Table 1). In day 10 cultures, about 4% of these 67 cells appeared to be lightly labelled by a 1 h BrdU pulse (Figure 2, Table 1). Few, if any, additional 3-day or 10-day 67 cells were labelled by a 36 h exposure to BrdU (Table 1) indicating that > 90% of these 67 cells would enter the Q-state after the next division (day 3 cultures) or were already in the Q-state (day 10 cultures). Although the percentage of proliferating cells in these day 10 cultures (≈6%) was slightly greater than that previously reported by Wallen et al (1984*a*), it is consistent with the shape and kinetics of the growth curve for 67 cells shown in Figure 1.

All $B_{max}$ values for the aneuploid 67 cells (Table 2) were slightly larger than the $B_{max}$ values previously reported for the diploid 66 cells (Mach et al, 1997). However, the 67 P- and 67 Q-cells were slightly smaller than the corresponding 66 P- and 66 Q-cells (Wallen et al, 1984*a*). Consequently, the slightly higher $B_{max}$ values for the 67 cells was offset by the smaller size of the 67 cells when the sigma-2 receptor density was calculated on a per cell basis. In these experiments, 3-day 67 P-cells had 300 000–800 000 sigma-2 receptors per cell (Table 1); a range identical to that previously reported for the diploid 66 P-cells (Mach et al, 1997). The 7- to 12-day Q-cells had 50 000–120 000 sigma-2 receptors per cell (Table 1); a range identical to that previously reported for the diploid 66 Q-cells (Mach et al, 1997). The P:Q ratio, expressed on a receptor per cell basis, was much greater than the $B_{max}$ P:Q ratio, because 67 P-cells had a higher protein content than 67 Q-cells (Table 2). The P:Q ratio, expressed on a receptor per cell basis, did not reach the maximum of ≈ 8 until day 10, even though > 90% of these 67 cells were in a quiescent state by day 7 (Figure 1, Table 1) (Wallen et al, 1984*a*). Although the number of sigma-2 receptors per P-cell, the number of sigma-2 receptors per Q-cell and the maximum P:Q ratio were identical for both the 66 and 67 cell lines, the 67 cells appeared to reach the maximum P:Q ratio slightly faster than the 66 cells (Figure 3). This is consistent with the 36 h BrdU labelling data in Table 1 which indicated that 3-day 67 cells underwent only one more division before the majority entered quiescent, while 3-day 66 cells underwent two divisions before the majority entered quiescence.

### Expression of sigma-2 receptors during the Q to P to Q transitions

When day 10 66 Q- and 67 Q-cells were trypsinized and reseeded at a concentration of $10^7$ cells per 175 cm$^2$ flask, the Q-cells rapidly entered the P-cell compartment with little or no delay (Figure 4). The kinetics of the expression of sigma-2 receptors in both 66 and 67 cells followed the population growth kinetics as expected (Figure 4). There was a rapid increase in the sigma-2 receptor density during the exponential growth phase that levelled off during early plateau phase and then decreased during late plateau phase. Although the kinetics of the expression of sigma-2 receptors followed the population growth kinetics, the decrease in the sigma-2 receptor density in late plateau phase did not start until day 10–12 after subculturing the Q-cells (Figure 4). Thus, the decrease in the sigma-2 receptor density occurred 3–5 days later if

© 1999 Cancer Research Campaign

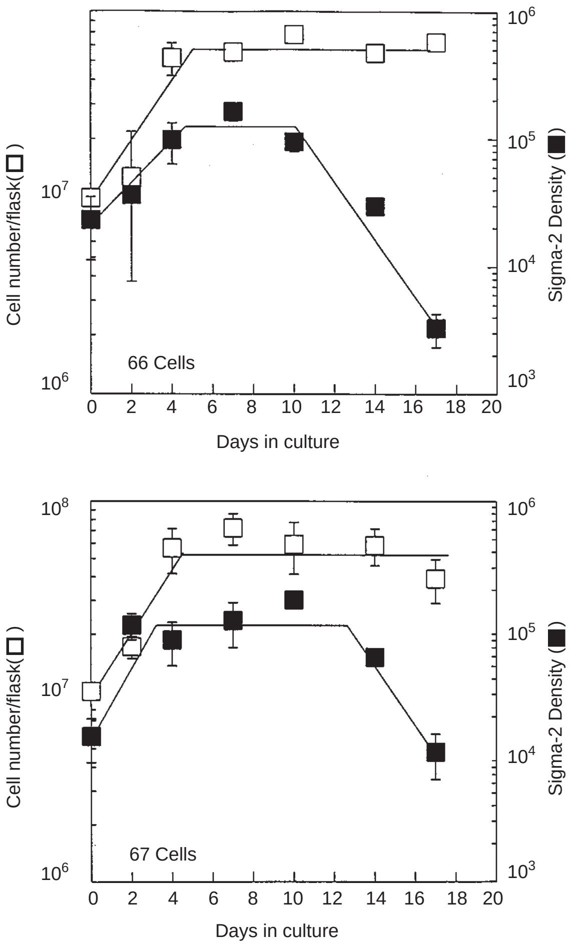

**Figure 4** Comparison of the population growth kinetics and the kinetics of expression of sigma-2 receptors when 10-day 66 Q-cells (upper panel) or 67 Q (lower panel) were recruited back into the P-cell compartment by subculturing. Each data point represents the mean ± 1 s.d. of the values from two independent experiments. If not shown, the error bars lie within the data point

**Table 3** Expression of sigma-2 receptors in exponentially growing and plateau phase 9L rat brain tumour cells

| Days in culture | $B_{max}$ (fmol mg$^{-1}$) (X ± 1 s.d.) | Receptors per cell (X ± 1 s.d.) |
|---|---|---|
| 3-day | 3239 ± 672 | 341 183 ± 22 263 |
| 5-day | 3200 ± 178 | 251 358 ± 23 568 |
| Exp:Plat | 1.01 ± 0.04 | 1.36 ± 0.08 |

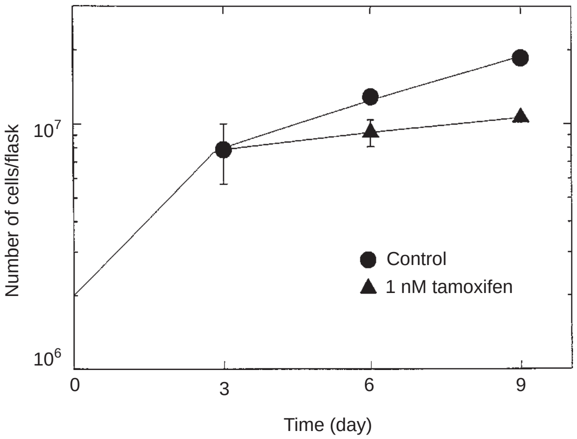

**Figure 5** In vitro growth curves for aneuploid MCF-7 human breast tumour cells with (▲) and without (●) continuous exposure to 1 nM tamoxifen starting on day 3 after subculturing from exponentially growing cells. Each data point represents the mean ± 1 s.d. of the cell counts from 2–3 independent experiments. If not shown, the error bars lie within the data point the cultures were set from 10-day Q-cells than if the cultures were set from 3-day exponentially growing P-cells.

### Effect of physiological and environmental factors on the expression of sigma-2 receptors

9L cells in 5-day cultures exist in medium that is virtually depleted of glucose and has a pH of 6.6–6.8 (Li, 1982*a*, 1982*b*; Zhang and Wheeler, 1994). These plateau phase cells grow in layers that are several cells thick and have a metabolism that is substantially reduced from that of exponentially growing cells (Wheeler et al, 1992). Finally, virtually the same number of cells labelled by a 1 h pulse of BrdU in day 3 and day 5 cultures (Table 1) indicates that day 4–6 plateau phase cultures result from an equilibrium between cell division and cell death as previously described (Wheeler and Wallen, 1980). The slight reduction in the number of cells labelled by a prolonged 36 h exposure to BrdU from 89% in 3-day cultures to 68% in 5-day cultures (Table 1) is also expected if the 9L plateau phase results from an equilibrium between cell division and cell death. Consequently, all of the physiological and environmental factors that might have modulated the sigma-2 receptor density of 66 and 67 cells in 7- to 12-day cultures are present in 5-day cultures of 9L cells; however, none of these 5-day 9L cells have entered a true quiescent state. The $B_{max}$ and the sigma-2 receptors per cell ratios for 3-day exponential and 5-day plateau phase 9L cells was 1.01 ± 0.04 and 1.36 ± 0.08 respectively (Table 3). For comparison, day 7 66 and 67 cells had exponential to plateau phase ratios of 2.8 (Mach et al, 1997) and 5.99 (Table 2) respectively. The slight decrease in the sigma-2 receptor density of the 5-day 9L cells (Table 3) probably occurred because a few of these 9L cells were clonogenically dead or dying. As described in Materials and Methods, non-clonogenic 9L cells have a lower sigma-2 receptor density than clonogenic 9L cells.

### Effect of cytotoxic concentrations of tamoxifen on the expression of sigma-2 receptors

Exposing day 3 cultures of oestrogen-responsive MCF-7 cells to 1 nM tamoxifen for either 3 or 6 days resulted in a virtual cessation of cell division (Figure 5). The number of MCF-7 cells recovered from the tamoxifen treated flasks on days 6 and 9 was not statistically different ($P > 0.05$) from the number of MCF-7 cells recovered from untreated flasks on day 3. By day 9, there was a 50% decrease in cell growth in the tamoxifen-treated flasks (Figure 5). No appreciable difference in the cell cycle distribution of the untreated or tamoxifen-treated cells was observed suggesting that few, if any of these MCF-7 cells, entered a true quiescent state (data not shown). However, there was a reduction in the number of sigma-2 receptors per cell after 3 or 6 days of treatment with 1 nM tamoxifen (Table 4). The average reduction in the sigma-2

© 1999 Cancer Research Campaign

**Table 4** Effect of tamoxifen on the expression of sigma-2 receptors in MCF-7 human breast tumour cells

| Days in culture | Experiments | Sigma-2 receptors per cell (untreated:treated) |
|---|---|---|
| 6-day[a] | A | 2.05 ± 0.11 |
| | B | 1.28 ± 0.11 |
| | C | 1.24 ± 0.13 |
| | | Average 1.52 ± 0.41 |
| 9-day[b] | B | 2.51 ± 0.33 |
| | C | 1.95 ± 0.28 |
| | | Average 2.23 ± 0.41 |

[a]Cells were treated with tamoxifen (1 nM) on day 3 after seeding. [b]Cells were treated with tamoxifen (1 nM) on day 3 and day 6 after seeding.

receptor density was 1.52 ± 0.41 after a 3-day exposure to 1 nM tamoxifen, and 2.23 ± 0.41 after a 6-day exposure to 1 nM tamoxifen. These values compare favourably with the reduction in the Ki-67 labelling index (1.24), AgNOR scores (1.32) and IdU–labelling index (2.36) reported by Dong et al (1997) for oestrogen-responsive MCF-7 cells whose growth had been reduced by 50% after exposure to 0.1–1.0 μM 4-hydroxy-tamoxifen.

### DISCUSSION

Sigma receptors were originally described as a subtype of the opiate receptors. Subsequent studies demonstrated that sigma receptors represented a distinct class found in a variety of tumours and normal tissues (Walker et al, 1990). Two types of sigma receptors have been identified. Sigma-1 receptors have a molecular weight of 25 kDa; sigma-2 receptors have a molecular weight of 18–21 kDa (Walker et al, 1990; Hellwell et al, 1994). In general, tumour cells have been reported to have more sigma-2 receptors than sigma-1 receptors (Bem et al, 1991; Vilner and Bowen, 1992; Vilner et al, 1995), and in at least one case, MCF-7 cells, it has been reported that sigma-1 receptors are undetectable (Vilner et al, 1995). Consequently, our studies have emphasized the establishment of a relationship between the density of sigma-2 receptors and the proliferative status of tumour cells because such a relationship is more likely to occur in a variety of human cancers.

At the present time, sigma-2 receptors have no known function, have no known endogenous ligand and have not been cloned. Our initial study with mouse mammary adenocarcinoma cells, line 66, indicated that proliferative tumour cells expressed about ten times more sigma-2 receptors per cell than quiescent tumour cells (Mach et al, 1997). In addition, the kinetics for the loss of sigma-2 receptors appeared to be identical to the kinetics for the loss of PCNA from these 66 Q-cells. Although these data suggested that sigma-2 receptors might be a biomarker of tumour cell proliferation, there were many biological, physiological and environmental factors other than progression through the cell cycle that could have been responsible for either the overexpression of sigma-2 receptors in 66 P-cells or the decreased expression of sigma-2 receptors in 66 Q-cells.

Among the biological reasons for the results with the 66 line was the possibility that the relationship between the expression of sigma-2 receptors and cell proliferation would be limited to diploid, rodent breast cancer lines. In addition, sigma-2 receptors might be a biomarker of the P to Q transition, but not of the Q to P transition. This would certainly limit the usefulness of sigma-2 receptors as a biomarker of tumour cell proliferation during and after therapy. There were also physiological and environmental factors that could have caused the apparent relationship between the expression of sigma-2 receptors and cell proliferation. For example, the metabolic status of these tumour cells could be drastically altered under nutrient depletion or low pH conditions, even though these cells may continue to progress through the cell cycle. If the expression of sigma-2 receptors reflected primarily changes in the metabolic state of the cells or the composition and pH of the surrounding milieu, sigma-2 receptors would not always be a biomarker of tumour cell proliferation.

In the study reported here, P-cells of the aneuploid mouse mammary adenocarcinoma line 67 had about eight times more sigma-2 receptors per cell than the Q-cells (Table 2); a result essentially identical to that previously reported for the diploid 66 line (Mach et al, 1997). Thus, ploidy did not appear to substantially influence the expression of sigma-2 receptors. This is particularly important if radioligands selective for sigma-2 receptors are going to be used to assess the proliferative status of human breast tumours because 25–30% of human breast tumours are comprised of predominantly aneuploid tumour cells, 25–30% are comprised of predominantly diploid tumour cells, and 30–40% contain substantial populations of both aneuploid and diploid tumour cells.

In many tissue culture models, there is a strong relationship between cell–cell contact, nutrient depletion, low pH, metabolic state and cell proliferation. In general, as cell–cell contact increases and nutrients are depleted from the medium, the pH, cell metabolism and cell proliferation decreases. 9L rat brain tumour cells are somewhat unique in that they will grow exponentially with their normal doubling time at pH 6.6–6.8, thereby forming a monolayer that is several cells thick (Zhang and Wheeler, 1994). As the glucose is depleted from the medium, the oxygen consumption of these 9L cells decreases and other sources of energy such as succinate are utilized if they are present (Wheeler et al, 1992). Although day 4 and day 5 9L cells have a distinctly altered metabolism, 9L cells continue to progress through the cell cycle at nearly a normal rate (Figure 2 and Table 1). When the glucose is sufficiently depleted (day 5–6), these 9L cells rapidly progress to death and lysis, rather than entering a quiescent state (Li, 1982*a*, 1982*b*). Death occurs at all phases of the cell cycle within 24 h, not just in M or G1. In the present study, day 5 9L cells which had existed at pH 6.6–6.8 for at least 48 h, were in a monolayer several cells thick, had a reduced metabolism from the day 3 cells, and existed at a glucose concentration that would result in the 60–80% normal clonogenic efficiency being reduced to < 1% within the next 48 h. None of these physiological or environmental factors resulted in a change in the sigma-2 receptor density from that found in day 3 exponentially growing 9L cells (Table 3). Consequently, this result suggests that cell–cell contact, nutrient depletion, low pH and altered metabolic states are not likely to be important regulators of the sigma-2 receptor density in tumour cells.

Although the 66, 67, and 9L data strongly support the hypothesis that the density of sigma-2 receptors in tumour cells reflects solely their proliferative status, the information will be useless unless the expression of the sigma-2 receptors matches changes in the proliferative status observed after treatment with various anti-cancer agents. It is well known that recruitment of Q-cells back into the P-cell compartment is a major event in the regrowth of a tumour after treatment. When both 66 and 67 Q-cells were recruited back into the P-cell compartment by subculturing, the

© 1999 Cancer Research Campaign sigma-2 receptor density increased rapidly, reached a maximum in 2–4 days, and then decreased after the cells entered quiescence with kinetics that were similar to those previously described for these same cells during the P to Q transition (Figure 4 and Table 2) (Mach et al, 1997). Thus, the kinetics of the expression of sigma-2 receptors matched the population growth kinetics when 66 and 67 Q-cells were recruited back into the P-cell compartment. It is also well known that many anticancer agents have cytostatic effects as well as cytotoxic effects. For example, at low concentrations of tamoxifen, human breast tumour cells stop progressing through the cell cycle or do so only very slowly (Katzenellenbogen et al, 1984). At high concentrations of tamoxifen, human breast tumour cells are killed (Katzenellenbogen et al, 1984). When MCF-7 human breast tumour cells were treated in our study with a cytostatic concentration of tamoxifen, the sigma-2 receptor density was reduced to the same extent (Table 4) as the Ki-67-labelling index, AgNOR scores and IdU-labelling index reported by Dong et al (1997). Taken together, these data suggest that the sigma-2 receptor density will likely reflect the proliferative status of human tumours before, during and after treatment with anticancer agents.

In summary, the hypothesis that the expression of sigma-2 receptors is predominantly influenced by a tumour cell's ability to progress through the cell cycle in a timely manner, and not by other biological, physiological, or environmental properties was tested by comparing: (a) the expression of sigma-2 receptors in diploid (66) and aneuploid (67) mouse mammary adenocarcinoma cells, (b) the expression of sigma-2 receptors in exponentially growing and plateau phase 9L rat brain tumour cells, (c) the kinetics of expression of sigma-2 receptors with the population growth kinetics obtained when 10-day 66 Q- or 67 Q-cells were recruited back into the P-cell compartment, and (d) the reduction of the expression of sigma-2 receptors with the reduction of other biomarkers of cell proliferation after treatment of MCF-7 human breast tumour cells with cytostatic concentrations of tamoxifen. It should be emphasized that the above tests of the hypothesis involved tumour cells from three species (mouse, rat and human), two tumour cell types (breast and brain) and tissue culture models where the proliferative status of the cells changed drastically, slightly, or not at all. In every case, the expression of the sigma-2 receptors solely reflected the proliferative status of the tumour cells. Consequently, all of the data support the hypothesis that the expression of sigma-2 receptors is predominantly influenced by a tumour cell's ability to progress through the cell cycle in a timely manner, and suggest that sigma-2 receptors may be a universal biomarker of cell proliferation. If the in vitro results reported here can be demonstrated in solid tumours, then it should be possible to non-invasively assess the proliferative status of tumours and normal tissues using radioligands that selectively bind to sigma-2 receptors.

### ACKNOWLEDGEMENTS

This work was supported by a grant from Mallinckrodt, Inc. and a pilot project grant from the Breast Cancer Program of the Comprehensive Cancer Center of Wake Forest University. Tissue culture medium and *Mycoplasma* testing were provided by the Tissue Culture Core Laboratory of the Comprehensive Cancer Center of Wake Forest University, which is supported in part by NIH Grant CA 12197. The flow cytometry analysis was performed in the University of Rochester Cancer Center Flow Cytometry Facility. We wish to thank C Wall for technical support and B Tyler for preparation of the manuscript.

### REFERENCES

Awwad H (1992) Unconventional fractionation studies and $T_{pot}$ correlation. *Semin Rad Oncol* **2**: 62–66

Begg AC, Hofland I, Van Glabbeke M and Horiot JC (1992) Predictive value of potential doubling time for radiotherapy of head and neck tumor patients: results from the EORTC cooperative trial 22851. *Semin Rad Oncol* **1**: 22–25

Begg AC (1995) The clinical status of $T_{pot}$ as a predictor? Or why no tempest in a $T_{pot}$! *Int J Radiat Oncol Biol Phys* **32**: 1539–1541

Bem WT, Thomas GE, Mamone JY, Homan SM, Levy BK, Johnson FE and Coscia CJ (1991) Overexpression of σ receptors in nonneural human tumours. *Cancer Res* **51**: 6558–6562

Berthois Y, Katzenellenbogen JA and Katzenellenbogen BS (1986) Phenol red in tissue culture media is a weak estrogen: implications concerning the study of estrogen-responsive cells in culture. *Proc Natl Acad Sci USA* **83**: 2496–2500

Bradford MM (1976) A rapid and sensitive method for the quantitation of microgram quantities of protein utilizing the principle of protein-dye binding. *Anal Biochem* **72**: 248–254

Chaval P, Courdi A, Gioanni J, Vallicioni J, Santini J and Demard F (1989) The labeling index: a prognostic factor in head and neck carcinoma. *Radiother Oncol* **14**: 231–237

Corro R, Giaretti W, Sanguinet G, Geido E, Orecehia R, Guenzi M, Margarino G, Bacigalupo A, Garaventa G, Barbieri M and Vitale V (1995) In vivo cell kinetics in head and neck squamous cell carcinomas predicts local control and helps guide radiotherapy regimen. *J Clin Oncol* **13**: 1843–1850

Dong H, Bertler C, Schneider E and Ritter MA (1997) Assessment of cell proliferation by AgNOR scores and Ki-67 labeling indices and a comparison with potential doubling times. *Cytometry* **28**: 280–288

Dressler LG (1993) DNA flow cytometry measurements and their clinical relevance in node-negative breast cancer. *Recent Results in Cancer Res* **127**: 61–69

Eckert RL and Katzenellenbogen BS (1982) Effects of estrogens and antiestrogens on estrogen receptor dynamics and the induction of progesterone receptor in MCF-7 human breast cancer cells. *Cancer Res* **42**: 139–144

Grogan TM, Lippman SM, Spier CM, Slymen DJ, Rybski JA, Rangle CS, Richter LC and Miller TP (1988) Independent prognostic significance of a nuclear proliferation antigen in diffuse large cell lymphomas as determined by the monoclonal antibody Ki-67. *Blood* **71**: 1157–1160

Haustermans K, Hofland I, Pottie G, Ramaekers M and Begg AC (1995) Can measurements of potential doubling time ($T_{pot}$) be compared between laboratories? A quality control study. *Cytometry* **19**: 154–163

Hedley DW, Shankey TV and Wheeless LL (1993*a*) DNA cytometry consensus conference. *Cytometry* **14**: 471

Hedley DW, Clark GM, Cornelisse CJ, Killander D, Kute T and Merkel D (1993*b*) Consensus review of the clinical utility of DNA cytometry in carcinoma of the breast. *Cytometry* **14**: 482–485

Hellwell SB, Bruce A, Feinstein G, Orringer J, Williams W and Bowen WD (1994) Rat liver and kidney contain high densities of $\sigma_1$ and $\sigma_2$ receptors: characterization by ligand binding and photoaffinity labeling. *Eur J Pharmacol Mol Pharmacol Sec* **268**: 9–18

Katzenellenbogen BS, Norman MJ, Eckert RL, Peltz SW and Mangel WF (1984) Bioactivities, estrogen receptor interactions, and plasminogen activator-inducing activities of tamoxifen and hydroxytamoxifen isomers in MCF-7 human breast cancer cells. *Cancer Res* **44**: 112–119

Li CKN (1982*a*) The glucose distribution in 9L rat brain multicell tumour spheroids and its effect on cell necrosis. *Cancer* **50**: 2066–2073

Li CKN (1982*b*) The role of glucose in the growth of 9L multitumour spheroids. *Cancer* **50**: 2074–2078

Mach RH, Smith CR, Al-Nabulsi I, Whirrett BR, Childers SR and Wheeler KT (1997) $\sigma_2$ receptors as potential biomarkers of proliferation in breast cancer. *Cancer Res* **57**: 156–161

McGuire WL and Dressler LG (1985) Emerging impact of flow cytometry in predicting recurrence and survival in breast cancer patients. *J Natl Cancer Inst* **75**: 405–410

Sarkaria JN, Fowler JF, Lindstrom MJ, Jordan VC and Mulcahy RT (1995) The decreased influence of overall treatment time on the response of human breast tumour xenografts following prolongation of the potential doubling time ($T_{pot}$). *Int J Radiat Oncol Biol Phys* **31**: 833–840

Shackney SE, McCormack GW and Cuchural GJ (1978) Growth rate patterns of solid tumours and their relation to responsiveness to therapy. An analytic review. *Ann Int Med* **89**: 107–121

 © 1999 Cancer Research Campaign

Thames HD, Peters LJ, Withers HR and Fletcher GH (1983) Accelerated fractionation vs hyperfractionation: rationale for several treatments per day. *Int J Radiat Oncol Biol Phys* **9**: 127–138

Tsang RW, Fyles AW, Kirkbride P, Levin W, Manchul LA, Milosevic MF, Rawlings GA, Banerjee D, Pintilie M and Wilson GD (1995) Proliferation measurements with flow cytometry $T_{pot}$ in cancer of the uterine cervix: correlation between two laboratories and preliminary clinical results. *Int J Radiat Oncol Biol Phys* **32**: 1319–1329

Vilner BJ and Bowen WD (1992) Characterization of sigma-like binding properties of NB41A3, S-20Y, and N1E-115 neuroblastoma, C6 glioma, and NG 108-15 neuroblastoma-glioma hybrid cells: further evidence for sigma-2 receptors. *In Multiple Sigma and PCP Receptor Ligands: Mechanisms for Neuromodulation and Neutroprotection*, JM Kamenka and EF Domino (eds.) pp 341–353. NPP Books: Ann Arbor, MI

Vilner BJ, John CS and Bowen WD (1995) Sigma-1 and sigma-2 receptors are expressed in a wide variety of human and rodent tumour cell lines. *Cancer Res* **55**: 408–413

Walker JM, Bowen WD, Walker FO, Matsumoto RR, De Costa B and Rice KR (1990) Sigma receptors: biology and function. *Pharmacol Rev* **42**: 355–402

Wallen CA, Michaelson SM and Wheeler KT (1980) Evidence for an unconventional radiosensitivity of rat 9L subcutaneous tumors. *Radiat Res* **84**: 529–541

Wallen CA, Higashikubo R and Dethlefsen LA (1984*a*) Murine mammary tumor cells in vitro. I. The development of a quiescent state. *Cell Tissue Kinet* **17**: 65–77

Wallen CA, Higashikubo R and Dethlefsen LA (1984*b*) Murine mammary tumor cells in vitro. II. Recruitment of quiescent cells. *Cell Tissue Kinet* **17**: 79–89

Wheeler KT and Wallen CA (1980) Is cell survival a determinant of the in situ response of 9L tumours? *Br J Cancer* **41**: Suppl. IV 299–303

Wheeler KT, Hickman R, Nelson GB, Moore SK and Wallen CA (1992) Relationship between DNA damage, DNA repair, metabolic state and cell lethality. *Radiat Environ Biophys* **31**: 101–115

Zhang H and Wheeler KT (1994) Radiation-induced DNA damage in tumors and normal tissues. II. Influence of dose, residual DNA damage and physiological factors in oxygenated cells. *Radiat Res* **140**: 321–326

*© 1999 Cancer Research Campaign*
